# Biomimetic strategies of cell membrane vesicles driven by pathogen-host interactions: novel insights into antimicrobial immunotherapy and infection prevention

**DOI:** 10.3389/fimmu.2025.1681889

**Published:** 2025-10-15

**Authors:** Ximin Xia, Yu Fang, Jia Zhong, Fanzhu Li, Lai Jiang

**Affiliations:** School of Pharmaceutical Sciences, Zhejiang Chinese Medical University, Hangzhou, China

**Keywords:** cell membrane vesicles, pathogen-host dynamic interaction networks, CMVs-derived formulations, bacterial immunomodulation, outer membrane vesicles, exosomes

## Abstract

Cell membrane vesicles (CMVs), including outer membrane vesicles from bacteria and exosomes from mammalian cells, have attracted growing attention due to their diverse roles in microbial pathogenesis and immune regulation. These vesicles not only facilitate the delivery of toxins and resistance factors during infection but also serve as critical messengers shaping host immune responses. In this review, we explore the bidirectional communication mediated by CMVs within pathogen-host networks, highlighting their influence on infection dynamics, immune evasion, and inflammation resolution. We further examine how these biological insights have inspired novel therapeutic strategies: engineered CMVs-based nanodecoys that block bacterial adhesion or neutralize virulence factors, and CMVs-derived nanoformulations that promote targeted immune activation. Recent advances in vesicle bioengineering have enhanced their targeting precision and immunogenic potential, offering promising solutions against antibiotic-resistant bacteria. Despite the progress, challenges related to production scalability, quality control, and regulatory approval remain significant. Continued interdisciplinary efforts are needed to advance CMVs-based technologies toward clinical application in infectious disease prevention and immune therapy.

## 1 Introduction

Cell membrane vesicles (CMVs) are spherical bilayer lipid structures secreted by prokaryotic and eukaryotic cells. They contain proteins, nucleic acids, lipids and metabolites, and the vesicle membrane is embedded with a variety of receptors, transporters and antigen-presenting molecules. This unique structure enables it to protect and deliver molecular cargo, and mediate communication between cells and even across species (1). The analysis in this review mainly focuses on two types of vesicles: (i) bacterial outer membrane vesicles (OMVs) and mammalian cell membrane vesicles.

In bacteria, outer membrane vesicles are typical representatives. They carry pathogen-associated molecules and virulence factors, and play an important role in the process of infection, immune escape and quorum sensing (2–5). Eukaryotic cells mainly communicate through vesicles via exosomes. Exosomes are generated by the multivesicular body pathway and contain a variety of proteins, RNA and metabolites. Their release increases significantly with stress states such as infection or tumors, thereby helping cells adapt to environmental changes (2–7). Exosomes are also highly plastic and can be surface-modified to enhance their stability and targeting, so they are considered a potential biological delivery platform (8).

With the deepening of research, CMVs are not only valued in basic biology, but also show significant translational potential. In the field of scientific research, they are important tools for understanding the molecular communication between pathogens and hosts; in clinical practice, they can be used as disease markers, diagnostic tools or delivery vectors (9). Through engineering modification, CMVs are expected to achieve functions such as neutralizing toxins, delivering antigens, and inducing specific immune responses (10–14). Their natural self-assembly properties also give them unique advantages in intelligent drug delivery and biosensor development. Compared with traditional antibiotic or monoclonal antibody therapy, the CMV-based strategy can not only directly fight against pathogens, but also regulate host immunity and microenvironment at the same time, providing the possibility of multi-dimensional intervention for complex infections. It is worth noting that the engineering modification of vesicles has produced a series of examples with translational potential. For example, the meningococcal OMV vaccine (Bexsero^®^) has been approved for clinical application; biomimetic nanovesicles can efficiently neutralize bacterial toxins; and the surface modification and molecular loading of exosomes have continuously expanded their applicability in immunotherapy. To more intuitively display these key advances, we summarized several representative examples of vesicle bioengineering in **Table 1**.

**Table 1:** Representative examples of vesicle bioengineering and their translational application prospects.

Vesicle type	Engineering strategy	Application field	Representative progress
Bacterial OMVs (Bexsero)	Prepared by combining recombinant proteins (such as fHbp, NadA and NHBA) with *Neisseria meningitidis* serogroup B OMV.	Prevention of invasive meningococcal disease caused by *Neisseria meningitidis*	It has been approved by the FDA and EMA and commercialized for use in immunization programs for children and young adults. Multiple clinical trials have confirmed its safety and efficacy, and it represents a successful example of OMV vaccine transformation.
Bacterial OMVs (Avacc 10)	Engineered bacterial OMVs (e.g., derived from *Neisseria* or *Vibrio cholerae*) as intranasal delivery platforms loaded with SARS-CoV-2 spike protein subunit antigens.	COVID-19 vaccine	Developed by Intravacc, the vaccine has completed Phase I clinical trials, with results showing good tolerance, safety and immunogenicity. The vaccine uses the modular characteristics of OMV, has the potential to quickly adapt to new variants, and is suitable for epidemic response.
Cell membrane vesicles	The macrophage membrane, red blood cell membrane and platelet membrane were wrapped on the surface of PLGA nanoparticles by membrane extrusion technology to obtain Nanosponges.	Bacterial toxin adsorption	The nanosponges effectively neutralized a variety of toxins from MRSA and demonstrated neutralizing toxicity in *in vitro* and *in vivo* experiments.
Immune cell membrane vesicles	The cell membrane vesicles of immune cells adsorb the toxins of bacteria to form nano-toxoids.	Vaccine	Nanotoxoids can rapidly and controllably induce host immune responses to resist bacterial invasion and improve the survival rate of mice.

In summary, CMVs are considered to be an important medium for the interaction between pathogens and hosts, and the mechanism of action in infection and immune regulation has attracted increasing attention. To better understand their biological significance and application prospects, this paper will further explore the role of CMVs in the “attack and defense game” between pathogens and hosts, and will explain in detail in the subsequent application-related content how these immune-related processes inspire the design of a new generation of biomimetic antibacterial immune strategies (immune decoys and antibacterial vaccines).

## 2 Pathogen-host dynamic interaction networks: the “offense-defense game” driven by CMVs

CMVs play a key role in microbial infections and immunological responses by acting as channels for communication between species and within species, which leads to complex signaling between pathogens and hosts.

### 2.1 Bidirectional information flow

First, CMVs let information flow in both directions. Bacteria use OMVs for “active offense”, mostly by sending virulence elements to specific places. Enterohemorrhagic *Escherichia coli* (EHEC) puts Shiga toxin, cytolethal distending toxin V, and EHEC-hemolysin into OMVs, which stops protein synthesis, damages DNA, and weakens the integrity of mitochondria (15). *Helicobacter pylori* (*H. pylori*) OMVs have VacA and CagA, which cause vacuolation and autophagy in host cells (16). Salmonella OMVs, on the other hand, have the genotoxin complex PltA-PltB-CdtB, which enters Caco-2 cells through dynamin-dependent endocytosis and cuts genomic DNA (17). The host has developed tiered defenses that focus on exosome-based counter-attacks in response to these many different types of attacks. When HaCaT keratinocytes take in *Staphylococcus aureus* (*S. aureus*) α-toxin, they don’t entirely break it down. Instead, they move the intact toxin into “toxosomes”, which are then released outside the cell, stopping pore creation (18). More specifically, endothelial cells that express ATG16L1 and are exposed to α-toxin release exosomes decorated with ADAM10 that attach to and trap the toxin, causing it to clump together and stop working, which slows the spread of the infection (19).

### 2.2 Reciprocal interactions

Second, vesicles change how pathogens and hosts interact with each other in both directions. This is an example of reciprocity: bacteria can avoid the immune system by using OMVs. *Haemophilus influenzae* adds the complement inhibitor Protein F to its OMVs, which stops the construction of the membrane-attack complex and the complement cascade. This makes the bacteria more resistant to serum and helps it colonize for a long time (20). *Pseudomonas aeruginosa* (*P. aeruginosa*) OMVs deliver short RNA52320, which greatly reduces the secretion of IL-8 from human bronchial epithelial cells and the recruitment of neutrophils. At the same time, this RNA52320 causes vitronectin to build up in alveolar epithelia, which helps the bacterium avoid being cleared by complement (21, 22). On the other hand, host exosomes fight back: Exosomes with antigens are effectively transmitted to antigen-presenting cells, which improves the presentation of the antigens and boosts T-cell activation, leading to strong adaptive immunity (23–25). In chronic infection, host exosomes change the immune milieu even more by carrying regulatory molecules that attract certain immune cells or change inflammatory mediators, which slows the growth of pathogens.

### 2.3 Temporal dynamics of infection

Third, vesicles change the timing of infection, which affects how the host and pathogen interact. Early in an infection, bacterial OMVs often include pro-inflammatory chemicals on them that make the host respond and summon in leukocytes. This makes areas where pathogens can spread more easily. Later, to minimize the harm caused by too much inflammation, certain OMVs move their cargo to anti-inflammatory lipids that dampen immune attacks (26). Host cells do the same thing: exosomes that transport TGF-β and IL-10 reduce inflammation and promotes repair (27, 28). In models of bacterial sepsis or acute lung injury, an early wave of EVs from the host carries pro-inflammatory signals. A later wave that is rich in miR-10a, miR-146a, and TGF-β/IL-10 polarizes macrophages toward an M2 reparative phenotype, stops NF-κB signaling, and accelerates tissue recovery. This determines whether clearance or coexistence happens (29).

### 2.4 Population-level communication

Lastly, vesicles have an effect on more than just individual pathogen-host pairs; they also affect communication within and between populations. OMVs distribute quorum-sensing molecules that control the creation of biofilms, the release of toxins, and the dissemination of antibiotic-resistance determinants within bacterial communities. This allows for the quick establishment of population-level resistance (30, 31). Host cells spread similar effects on populations: When *Salmonella Typhimurium* (*S. Typhimurium*) infects RAW264.7 and bone-marrow-derived macrophages, they produce exosomes that activate naïve BMDMs and BMDCs at distant regions, causing them to release TNF-α and IL-6 (32). When THP-1 cells are exposed to *Mycobacterium bovis* BCG or *S. Typhimurium*, they make exosomes that contain LPS. These exosomes turn on p38 and IκBα through TLR4/MyD88 and increase TNF-α production (33). Host exosomes that carry immune-modulating chemicals interact with the gut microbiota, keeping it diverse and stopping diseases that are caused by dysbiosis. On the other hand, OMVs from friendly bacteria send metabolites and signaling molecules that help epithelial cells tolerate the resident flora, which reduces the immune system’s overreaction to it (34, 35). This two-way modulation keeps a healthy host-microbe relationship and gives us new ideas on how to treat complicated infections.

## 3 Innovation and expansion of CMVs-derived formulations in bacterial immunomodulation

CMVs act as encrypted channels for the constant flow of information between pathogens and hosts. They show co-evolutionary strategies and also provide a model for the future generation of antimicrobials. There is currently a unique niche in bacterial immunomodulation for vesicle-inspired formulations that combine the innate immunogenicity of CMVs with the accuracy of synthetic engineering. These constructions can be filled with pathogen-associated molecular patterns to activate host pattern-recognition receptors, which starts strong innate responses. Changes to the surface or lumen make antigen delivery even better, directing adaptive immunity with excellent specificity. Their functional architecture is important because it allows them to do two things: operate as decoys that trap released virulence factors and stop the spread of infection, and act as immunotools that present pathogen-derived antigens to trigger specific immune activation. This bifunctional architecture gives us a brand-new way to regulate the immune system’s ability to fight off infections, especially when it comes to multidrug-resistant bacteria and complicated infectious situations where traditional methods don’t work.

### 3.1 Applications of the decoy strategy

At the heart of the decoy strategy, CMVs-derived formulations recapitulate the architecture and molecular repertoire of host cells. By engaging bacterial virulence factors at the pathogen-host interface, these vesicles absorb secreted toxins or sterically hinder microbial adhesion, thereby shielding native tissues from direct injury. Membranes harvested from erythrocytes or macrophages exhibit intrinsic avidity for bacterial toxins and pathogenic ligands, endowing the resulting formulations with an evolutionarily refined affinity that can be exploited without further molecular engineering.

#### 3.1.1 Precision interception of bacterial colonization

**Figure 1:**
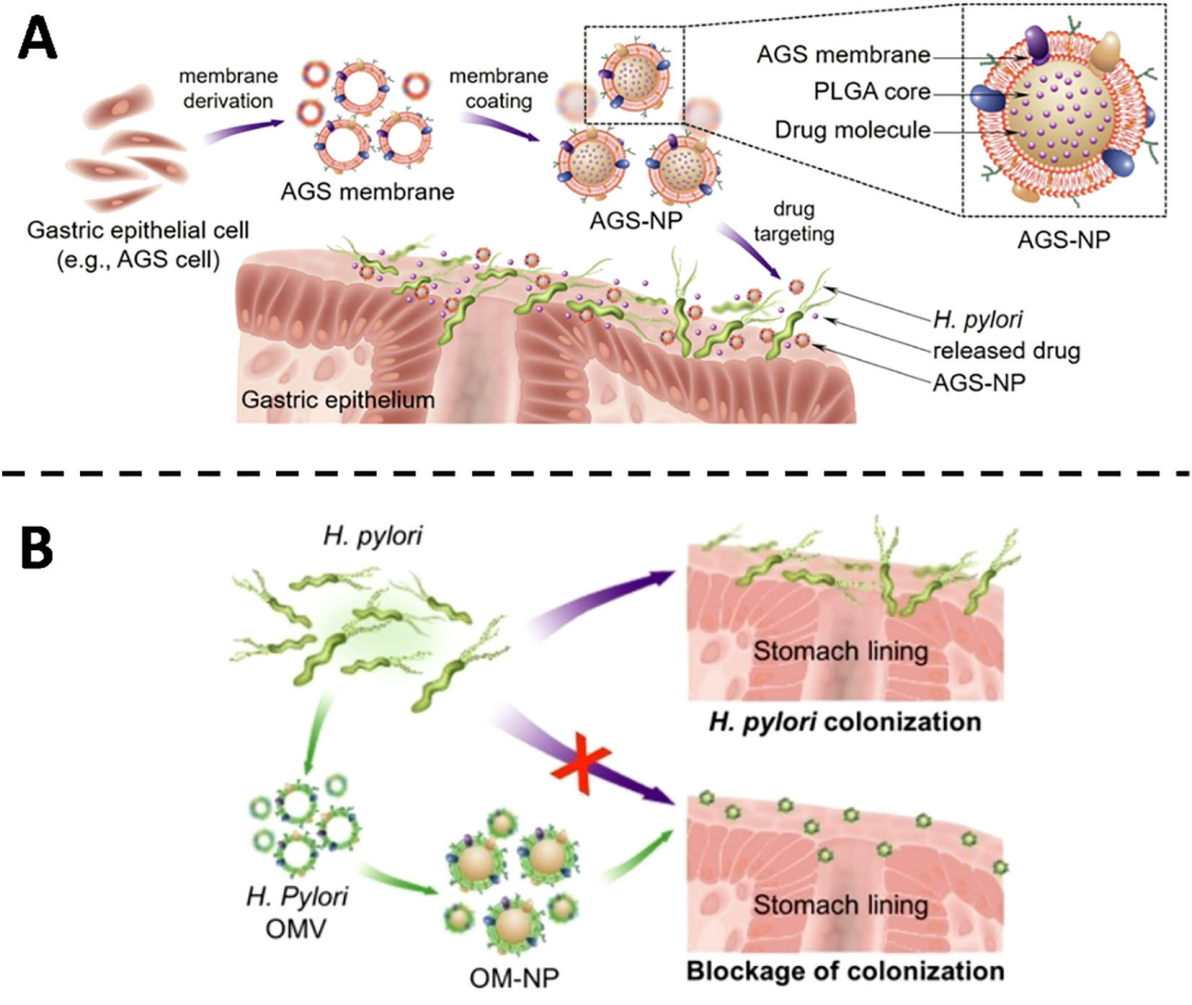
**(A)** Illustrative diagram showing the fabrication of AGS-NPs and their application in targeted antibiotic delivery for combating *H*. *pylori* infection. Copyright ^©^ 2018 Wiley-VCH Verlag GmbH & Co. KGaA, Weinheim **(B)** Schematic overview showing the anti-adhesive function of OM-NPs against *H*. *pylori* on the stomach epithelium. Copyright ^©^ 2019 Wiley-VCH Verlag GmbH & Co. KGaA, Weinheim.

Pathogen tethering to host surfaces is the decisive first step in colonization and subsequent invasion. Membrane-cloaked nanodecoys exploit this obligatory interaction by presenting cognate receptors on a synthetic scaffold, sequestering the organism before it can anchor to native tissue. For *H. pylori*, gastric colonization is contingent upon high-affinity binding to epithelial receptors such as integrin β1, fucosylated Lewis blood-group antigens and carcinoembryonic antigen-related cell-adhesion molecules. Capitalizing on this tropism, Angsantikul and colleagues (36) harvested plasma membranes from human gastric epithelial AGS cells and enrobed a polymer core loaded with clarithromycin (**Figure 1A**). The resulting AGS-NPs inherit the surface landscape of their parental cells and therefore engage *H. pylori* with native avidity. *In vitro*, the particles accumulate preferentially on bacterial surfaces; in murine infection models, clarithromycin-loaded AGS-NPs outperform both free drug and non-targeted nanoparticles, markedly suppressing colonization without off-target exposure. Zhang et al. (37) advanced an orthogonal anti-adhesion paradigm. Instead of host-derived membranes, they enveloped a polymeric core with outer-membrane vesicles isolated from *H. pylori* itself, yielding OM-NPs that mirror the bacterial surface (**Figure 1B**). These decoys compete directly with viable organisms for the same epithelial receptors, thereby reducing attachment in a concentration- and sequence-dependent manner. Both strategies leverage membrane-mediated recognition to interrupt colonization, yet they differ fundamentally in origin-host membrane for targeted drug delivery versus bacterial membrane for competitive exclusion. Collectively, such biomimetic constructs illustrate a versatile approach to circumvent antimicrobial resistance by preventing, rather than merely killing, bacterial establishment.

#### 3.1.2 Efficient neutralization of bacterial toxins

**Figure 2:**
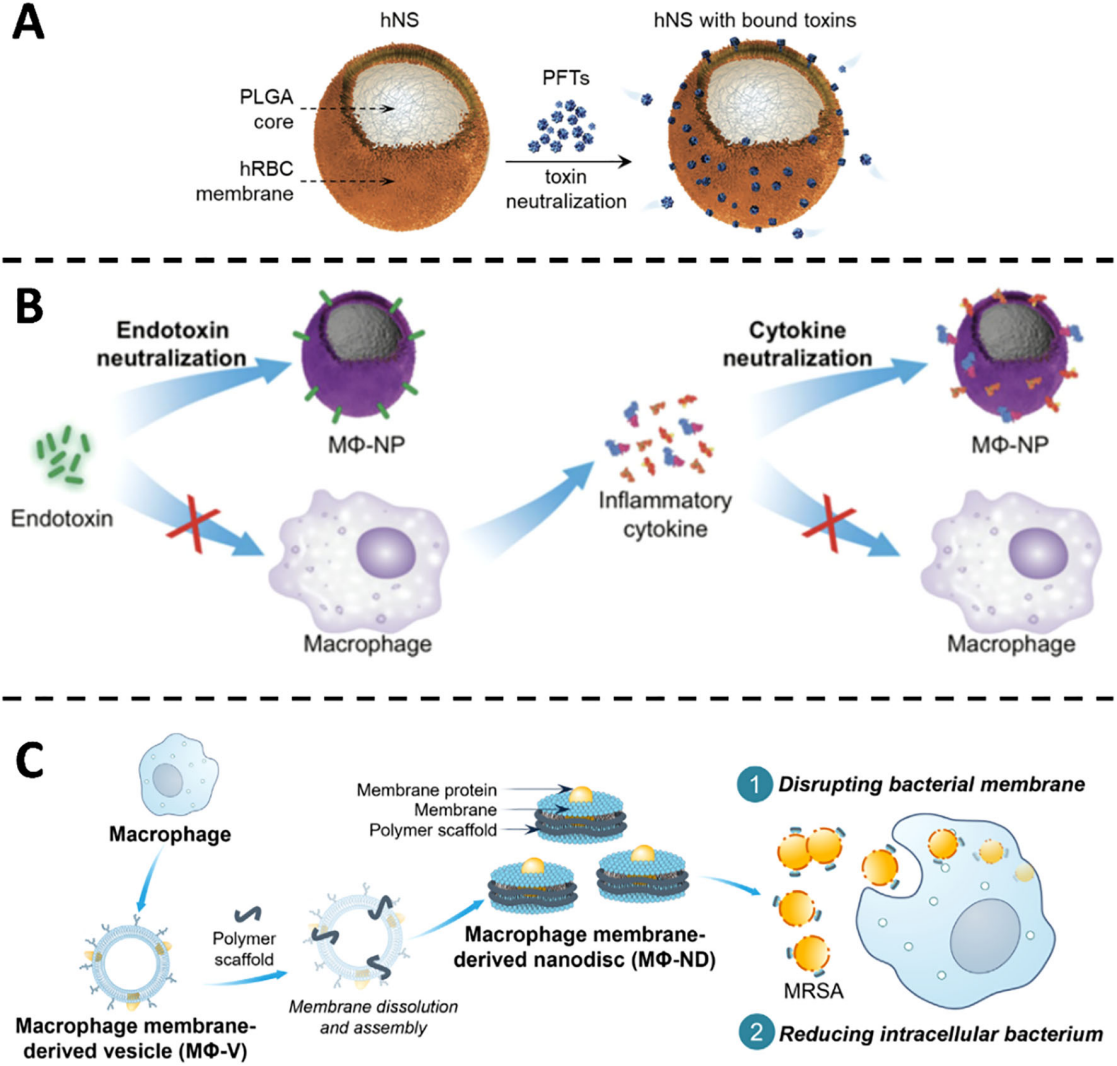
**(A)** Illustration of hNS design and its functional mechanism in blocking pore-forming toxins. Copyright ^©^ 2018 Wiley-VCH Verlag GmbH & Co. KGaA, Weinheim **(B)** Visual depiction of MΦ-NPs acting in a two-phase manner to counter endotoxins and cytokine storms during sepsis. Copyright ^©^ 2017 Thamphiwatana et al., PNAS. Adapted with permission under a License CC BY-NC-ND **(C)** Illustrative overview of how MΦ-NDs are produced and combat MRSA by damaging membranes and diminishing internalized bacteria. Copyright ^©^ 2025 Feng et al., Science Advances. Adapted with permission under a License CC BY-NC.

CMVs-derived formulations emulate the surface microenvironment of host cells, sequestering bacterial toxins before they can inflict membrane damage. The strategy hinges on the high-affinity, lock-and-key interaction between membrane-embedded proteins and soluble virulence factors, thereby neutralizing the toxins at the extracellular phase. In *S. aureus* infection, the pore-forming α-toxin lyses host cell membranes and precipitates local tissue injury. Nanoparticles cloaked with erythrocyte membranes act as molecular sponges that adsorb α-toxin and prevent its insertion into target bilayers. In a seminal study, Hu et al. generated RBC membrane-coated polymeric nanoparticles that diverted membrane-damaging toxins away from cellular targets (13). In murine sepsis models, the nanosponges markedly attenuated α-hemolysin toxicity and significantly improved survival. Building on this foundation, Chen (38) (**Figure 2A**), Zhang (39) and Ben-Akiva (40) extended the concept to a spectrum of bacterial toxins. Erythrocyte-membrane vesicles were shown to neutralize α-hemolysin from both methicillin-sensitive and methicillin-resistant *S. aureus* (MSSA and MRSA), listeriolysin O from *Listeria monocytogenes* and streptolysin O from *Streptococcus pyogenes*. *In vitro*, toxin-induced haemolysis was abolished in a concentration-dependent manner; adsorbed toxins exhibited no residual cytotoxicity or lethality in cell culture or *in vivo* assays. Beyond pore-forming toxins, ϵ-toxin from *Clostridium perfringens* was effectively intercepted by erythrocyte-camouflaged nanoparticles (41). Whether administered intravenously or delivered via aerosol, the constructs provided time-dependent protection against ϵ-toxin challenge, underscoring the broad utility of red-cell-membrane-based nanodecoys as versatile anti-toxicity platforms.

Thamphiwatana and colleagues encapsulated polymeric nanoparticles with macrophage-derived membranes, thereby reconstituting the native antigenic landscape of the source cell (**Figure 2B**). The resulting nanoparticles sequester endotoxin and inflammatory cytokines, blunt pathogen-associated molecular pattern (PAMP) recognition, and interrupt the escalating cytokine cascade characteristic of Gram-negative bacteremia. In a murine model of *E. coli* sepsis, administration of these macrophage-mimetic particles reduced systemic pro-inflammatory mediators, restrained bacterial dissemination, and doubled survival relative to untreated controls (14). Liu et al. extended this paradigm by grafting macrophage-membrane vesicles onto silicon nanowire arrays integrated within a microfluidic cartridge. Ex vivo perfusion of septic human blood through this device removed >90% of circulating endotoxin and cytokines within four hours, outperforming conventional hemofiltration. When the same cartridge was employed for extracorporeal blood cleansing in septic mice, cytokine levels normalized and survival increased four-fold, underscoring the superior detoxifying capacity of membrane-camouflaged nanowires (42). Recently, Feng and co-workers fabricated macrophage-membrane nanodiscs (MΦ-NDs) that neutralize pore-forming toxins produced by MRSA (**Figure 2C**). These discoidal vesicles adsorb α-toxin with nanomolar avidity, disrupt bacterial membranes, and simultaneously potentiate macrophage-mediated bacterial clearance. In a lethal MRSA challenge model, MΦ-NDs conferred 100% survival without discernible toxicity and elicited no detectable resistance, features that highlight their translational promise (43).

Building on these precedents, Jing et al. engineered ultrasound-responsive asymmetric polymeric microbowls armored with hybrid macrophage and erythrocyte membranes. Under acoustic fields, the microbowls execute contactless, programmable trajectories that accelerate mass transfer within physiological fluids (44). Within fifteen minutes, the constructs removed 92.8% of endotoxin; erythrocyte-membrane domains simultaneously sequestered Pb²^+^ and Hg²^+^ with efficiencies of 92.75% and 93.91%, respectively. Macrophage-membrane proteins mediated selective adhesion and eradication of >90% of *S. aureus*, demonstrating broad-spectrum pathogen captured in a single platform.

Departing from passive adsorption, Pang et al. genetically engineered membrane nanovesicles to display monoclonal antibodies against α-toxin, thereby shifting from broad-spectrum scavenging to precision toxin neutralization. The antibody-decorated vesicles exhibited picomolar affinity for α-toxin and, when conjugated with sonosensitizers, generated reactive oxygen species under ultrasound excitation to augment both antibacterial and antitoxic efficacy (45). Collectively, these studies trace an arc from natural-membrane mimicry to gene-edited, stimulus-responsive therapeutics, illustrating the expanding technological repertoire and clinical potential of membrane-vesicle-based detoxification strategies.

To enhance the rational design of decoy vesicles, it is essential to delineate many universal criteria. The membrane composition dictates the specificity of toxin binding: for instance, the erythrocyte membrane, abundant in cholesterol and glycoproteins, effectively captures pore-forming toxins; conversely, the macrophage membrane features diverse pattern recognition receptors (PRRs), which are more adept at neutralizing endotoxins or inflammatory mediators. Secondly, the membrane’s fluidity influences the accessibility and binding kinetics of the receptor, while the density of the membrane protein dictates the overall adsorption capacity. Moreover, the dimensions and curvature of vesicles, along with other structural characteristics, will further influence the binding affinity and phagocytic route of toxins. Cell membranes derived from various sources possess distinct advantages for particular applications. Biomimetic vesicles originating from red blood cell membranes are effective in neutralizing hemolytic toxins; vesicles from macrophage membranes exhibit a wider range of protective effects by concurrently adsorbing endotoxins and inflammatory mediators; platelet membranes selectively engage with bacterial adhesion factors; and tumor cell membranes may provide immune regulatory functions. The aforementioned comparison indicates that the source, composition, and physicochemical characteristics of the membrane collectively influence the neutralizing efficacy of the decoy vesicles.

### 3.2 Applications for the immunotherapeutic strategy

Leveraging their intrinsic biomimicry and potent immunomodulatory capacity, CMVs-inspired nanoformulations are rapidly emerging as a versatile platform for antimicrobial immunotherapy. Through rational design, these vesicles faithfully recapitulate the molecular landscape of native membranes while accommodating additional functionalities via orthogonal engineering. By orchestrating the spatiotemporal delivery of antigens or immunoregulatory cues, they synchronize humoral and cellular effector arms, thereby amplifying the overall efficacy of host defense against invading pathogens.

#### 3.2.1 Bioengineering advances are accelerating the evolution of native bacterial OMVs into next-generation vaccines

Native OMVs inherently display a broad repertoire of pathogen-associated antigens and faithfully reproduce the membrane interactions that occur between bacteria and host cells. Through this biomimetic engagement, OMVs efficiently activate immunity: binding to host Toll-like receptors (TLRs) initiate inflammatory signaling cascades, promotes maturation of macrophages and dendritic cells, and elicits robust innate responses. This early activation not only fortifies the host’s first line of defense but also provides the critical priming signals required for adaptive immunity, markedly enhancing B-cell antibody production and T-cell-mediated cytotoxicity. It is precisely this convergence of membrane-mimetic recognition with dual innate and adaptive immunomodulatory capacity that endows OMVs with exceptional promise in antibacterial vaccine development. Comprehensive reviews of unmodified OMVs as prophylactic agents are already available (46–48); therefore, the present discussion focuses on recent bioengineering strategies that functionalize native OMVs to augment immunogenicity and antigen-delivery efficiency, while fully preserving the parental bacterial antigenic signature, thereby affording superior protective efficacy in pre-clinical models.

Baker and colleagues cultured *Burkholderia pseudomallei* under macrophage phagocytosis-mimetic conditions, yielding OMVs (termed M9-OMVs) enriched in intracellular-survival proteins. These vesicles were devoid of cytotoxicity and, after intranasal administration to mice, provided lung protection equivalent to that achieved with live-attenuated vaccine while eliciting robust IgG, CD4^+^ and CD8^+^ T-cell responses; DC maturation was likewise pronounced, underscoring intrinsic adjuvanticity (49). Huang et al. coated *Bordetella bronchiseptica* OMVs with pegylated rehmannan polysaccharide (pRL), forming concentric nanocomposites. Functional assays and transcriptomic analyses revealed heightened DC proliferation, cytokine release and phagocytosis, together with superior lymph-node accumulation. The transcriptional signature pointed to T-cell-receptor signaling and balanced Th1/Th2/Th17 polarization, confirming that pRL conjugation amplifies immunity without compromising parental antigens (50). Majumder employed an attenuated *Yersinia pseudotuberculosis* strain (YptbS46) engineered to express pneumococcal surface protein A (PspA) within OMVs (OMV-PspA). Coadministration with monophosphoryl lipid A induced durable memory responses and conferred complete protection against influenza-triggered secondary *Streptococcus pneumoniae* (*S. pneumoniae*) infection, including cross-protection against disparate pneumococcal serotypes (51). Jones and co-workers removed RmpM and PorB-two *Neisseria gonorrhoeae* proteins implicated in blocking antibody or immunosuppressive effects, and replaced PorB with its meningococcal counterpart. The resulting OMVs evoked markedly higher antigen-specific IgG and a Th1-skewed cytokine profile, demonstrating that strategic genomic editing can override native immune evasion mechanisms (52).

**Figure 3:**
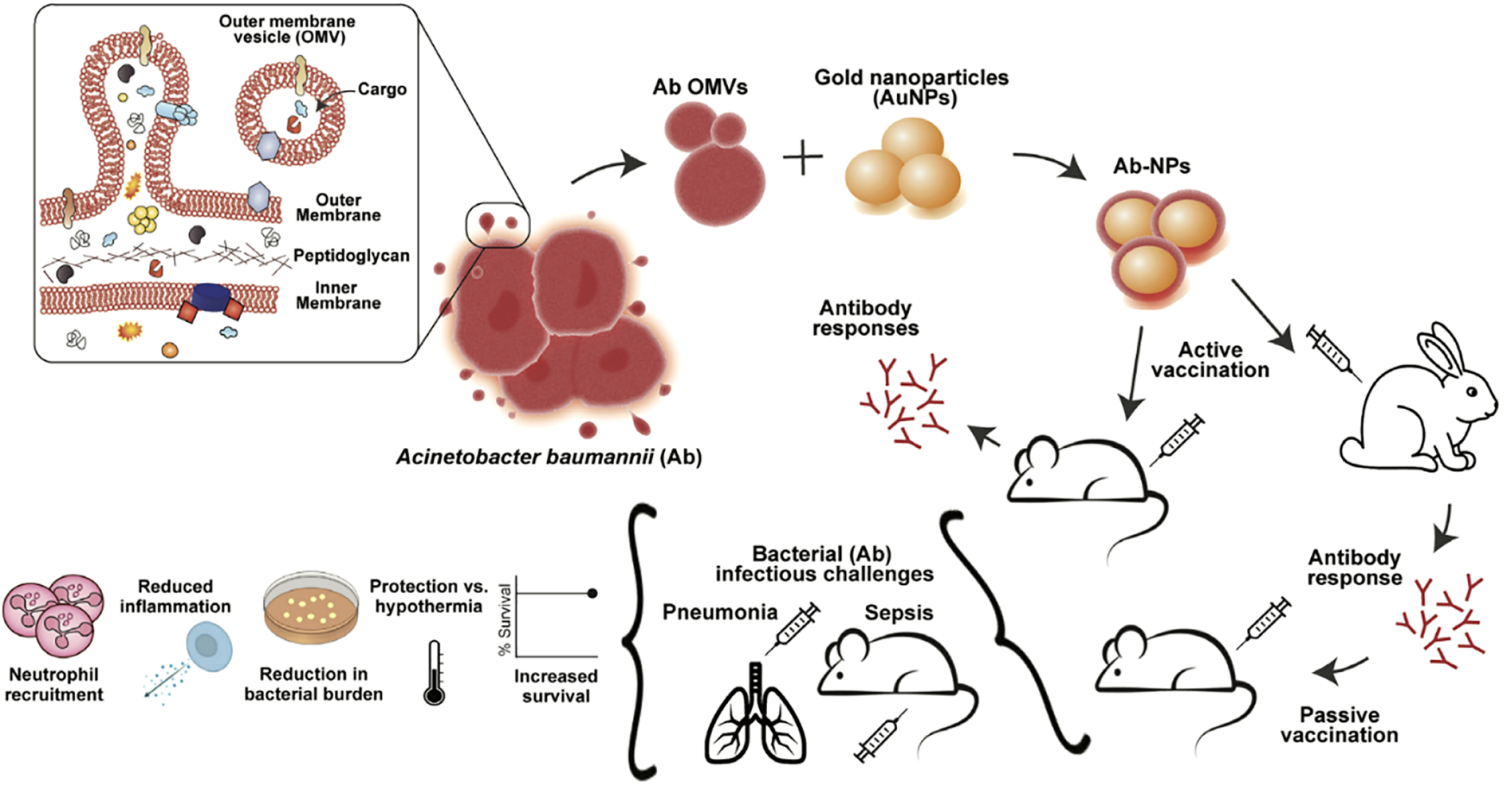
OMVs from *A. baumannii* Lac-4 were linked to gold nanoparticles to form Ab-NPs. This vaccine triggered specific immune responses, protected against pneumonia and sepsis, lowered inflammation, and limited bacterial spread. Copyright ^©^ 2022 Bjanes et al. Advanced NanoBiomed Research published by Wiley-VCH GmbH.

Moreover, integrating OMVs with nanomaterials markedly improves their stability and delivery performance, thereby extending their utility within intricate immunological milieus. Guo et al. anchored cytoplasmic and double-membrane vesicle antigens from multidrug-resistant *P. aeruginosa* PAO1 onto mesoporous silica nanospheres (MSN-BL@DMV) (53). The core-shell nanovaccine was avidly internalized by DCs in the absence of exogenous adjuvant, trafficked to draining lymph nodes, and engendered vigorous humoral and cellular immunity that protected mice against both homologous PAO1 and heterologous strain PA-XN-1. Bjanes and colleagues fused *Acinetobacter baumannii* (*A. baumannii*) OMVs to gold nanoparticles (Ab-NPs) (**Figure 3**). Immunization of rabbits generated high-titer IgG that potentiated human neutrophil phagocytosis; mice receiving the conjugate survived lethal *A. baumannii* sepsis. Mechanistically, Ab-NP-OMVs promoted B-cell homing to lymph nodes, up-regulated DC activation markers, enhanced splenic neutrophil effector functions and attenuated systemic cytokine release (54).

Taken together, these independent but convergent studies establish OMV-based platforms as versatile vaccines that retain the full antigenic identity of the parent bacterium while permitting modular enhancements for potency, breadth and durability, attributes urgently needed in the fight against multidrug-resistant pathogens.

#### 3.2.2 A neo-toxoid vaccine paradigm built on immune cell membrane interfacial crosstalk

Inspired by the intimate interplay between pathogens and host cells, immune cell membrane-based toxoid-mimetic vaccines are emerging as a distinct immunological modality. These constructs absorb bacterial toxins onto engineered immune cell membranes, simultaneously neutralizing cytotoxicity and preserving antigenic integrity, thereby converting a virulence factor into a potent immune-stimulus directed against the offending pathogen. This bifunctionality offers a decisive advantage in preventing or treating toxin-mediated infections caused by highly virulent bacteria.

**Figure 4:**
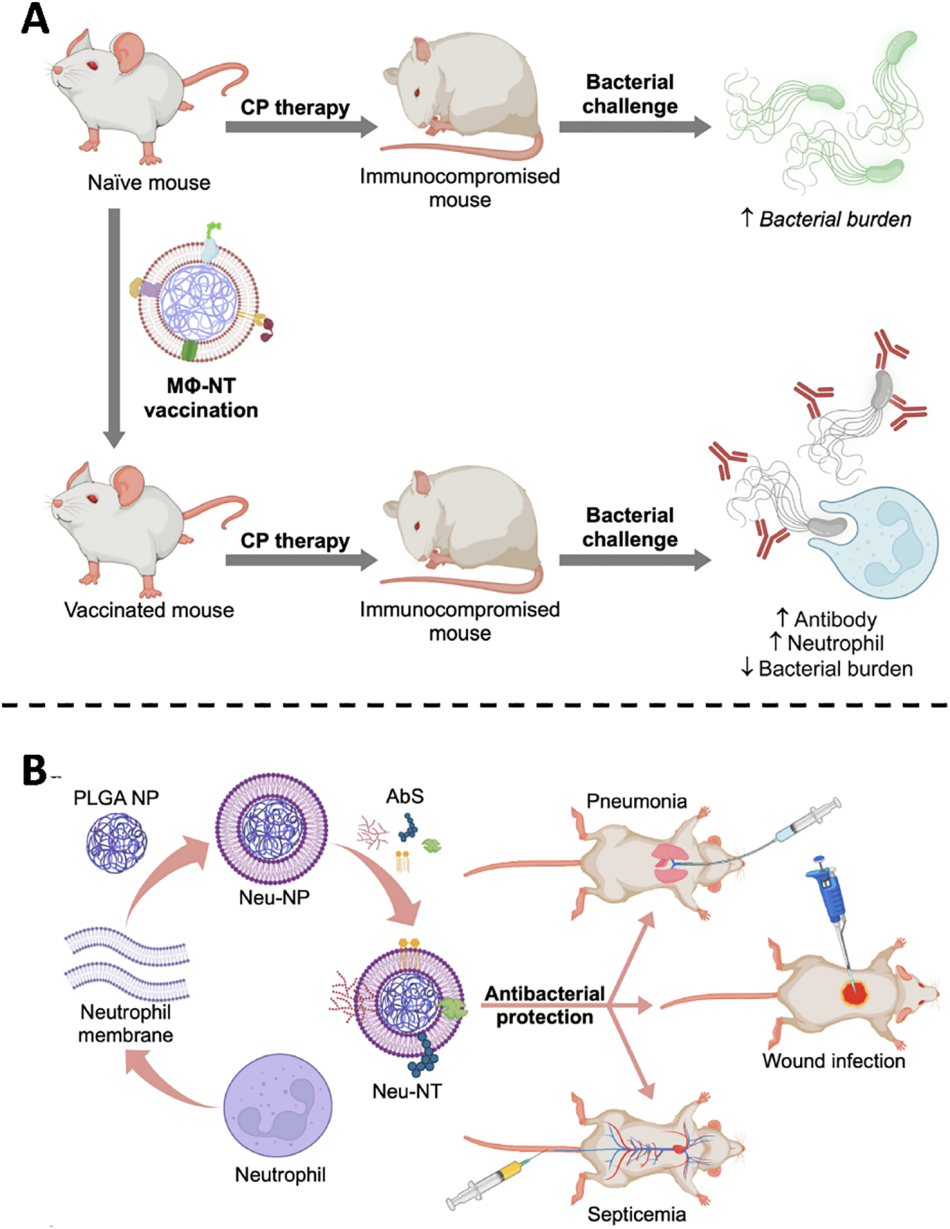
**(A)** MΦNT vaccination restores antibacterial defense in CP-immunosuppressed hosts by combining antibody-mediated recognition with enhanced neutrophil activity. Copyright ^©^ 2022 Zhou et al., Science Advances. Adapted with permission under a License CC BY-NC. **(B)** Neu-NTs, formed by loading **(A)** *baumannii* secreted proteins onto neutrophil-mimicking nanoparticles, confer protection in pneumonia, sepsis, and wound infection models. Copyright ^©^ 2022 American Chemical Society.

The Zhang laboratory has systematically advanced this concept through a succession of proof-of-principle studies. By anchoring bacterial toxins onto macrophage or neutrophil membranes, the group recapitulates pathogen-host recognition events while shielding tissues from damage. In an archetypal study, Zhou et al. encapsulated *P. aeruginosa* virulence determinants within macrophage-membrane vesicles (**Figure 4A**). Administration to immunocompromised animals elicited rapid, durable protection: toxin-neutralizing antibodies rose markedly, neutrophil-mediated killing was augmented, and lethal challenge was averted without observable adverse effects (55). Extending the platform to multidrug resistant *A. baumannii*, the same team engineered neutrophil membrane-coated nanoparticles that sequestered *A. baumannii* toxins while maintaining native conformational epitopes. Across murine pneumonia, sepsis and wound-infection models, vaccination significantly improved survival, curtailed pathogen dissemination and induced pathogen-specific immunity, thereby providing a versatile countermeasure against a notorious nosocomial pathogen (**Figure 4B**) (56).

The versatility of these formulations is underpinned by programmable design variables-toxin load, membrane source and surface chemistries-that can be tuned for precision targeting of disparate pathogens. Coupling with adjuvants or immunomodulators is expected to further amplify their potency, broadening utility within complex infectious landscapes. Continued advances in nanofabrication and translational pharmacology position immune membrane toxoid mimics not only as next-generation antibacterial vaccines, but also as adaptable platforms for viral, autoimmune and oncological immunotherapy.

## 4 Perspectives and challenges

Compared to conventional antibiotics and monoclonal antibodies, CMVs-based strategies offer distinct advantages: (1) dual functionality as decoys and immunomodulators, (2) ability to target both pathogens and host microenvironments, and (3) reduced risk of resistance development due to multi-mechanistic actions. As engineering strategies for membrane-mimetic vesicles evolve, their distinctive advantages in targeted delivery and immune modulation are becoming increasingly evident, positioning them as a central therapeutic platform for bacterial infections, immune dysregulation and related disorders. Current CMVs-derived formulations already recapitulate basic targeting and regulatory functions; the next generation must, however, achieve higher precision and adaptability. Three directions warrant priority. First, multidimensional targeting. Single-ligand decoration is insufficient for the heterogeneity inherent in complex infections. Insertion of “dual-recognition” cassettes-simultaneously engaging capsular polysaccharides and inflammation-associated epitopes-will enable pathogen-plus-microenvironment locking, thereby sparing healthy tissue. Second, environmental responsiveness. Stimuli-responsive polymers will confer “smart” behavior, allowing vesicles to release payloads exclusively within the acidified, enzyme-rich milieu of infected foci. Such spatiotemporal control mitigates premature degradation and widens the therapeutic window. Third, integrated multifunctionality. Modular assembly will merge targeting, therapeutic and diagnostic moieties within a single architecture, enabling real-time feedback on treatment efficacy.

Beyond anti-infective applications, the biocompatibility, immunomodulatory capacity and tropism of these CMVs-derived formulations invite broader exploitation. In chronic *P. aeruginosa* infection complicating cystic fibrosis, vesicles could ferry microbiota-modulating factors to restore pulmonary ecological balance and reduce relapse risk. For emerging infectious threats, antigen-decorated vesicles-bearing, for example, SARS-CoV-2 spike or Plasmodium merozoite proteins-could serve as rapid response mucosal vaccines, leveraging intrinsic adjuvanticity and epithelial-targeting ligands to enhance oral or intranasal uptake.

Despite encouraging pre-clinical data, the clinical translation of CMVs-derived formulations remains contingent upon a systematic, end-to-end development pipeline. The meningococcal outer-membrane vesicle vaccine 4CMenB (Bexsero^®^) exemplifies early success: its OMV fraction supplies native antigens, while recombinant factor H-binding protein (fHbp) broadens strain coverage, and the product has already secured pediatric approval (57). Yet such achievements are the exception rather than the norm.

Key challenges include the following four aspects:

1. Production and quality control are a major bottleneck. Natural bacterial outer membrane vesicles require large-scale, tightly controlled fermentation processes, which are often low-yielding and have batch-to-batch differences in purity and potency. Both cell membrane-coated synthetic vesicles and fully synthetic vesicles rely on laborious ultracentrifugation or microfluidic processing, which not only increases costs but also makes standardized production challenging. To address these limitations, a continuous flow system based on microfluidic chips needs to be developed to achieve high-yield preparation of uniform vesicles, combined with orthogonal quality control indicators based on mass spectrometry and multi-parameter flow cytometry.
2. The immunogenicity and safety risks cannot be ignored. Natural OMVs contain abundant pathogen-associated molecular patterns, which may induce systemic inflammatory reactions or even sepsis-like syndrome if they are present in excessive amounts. At the same time, toxins, hemolysins and nucleic acid fragments commonly found in vesicles may increase cytotoxicity or promote the spread of drug-resistant genes. Although synthetic vesicles can avoid some pathogenic components, they often accumulate in the liver and spleen *in vivo*, causing a long-term burden on the reticuloendothelial system. Therefore, it is imperative to establish a systematic safety assessment file, which can predict its *in vivo* distribution, metabolism and long-term toxicity with the help of humanized organoid models and long-term tracking experiments.
3. The potential off-target effect is also an important challenge. CMVs are rich in a variety of membrane receptors and adhesion molecules, which may bind to non-target cells, triggering non-specific immune responses or interfering with normal signaling pathways. Recent studies have attempted to reduce off-target effects by surface modification (such as PEGylation or targeted ligand modification) and optimization of administration routes (such as local delivery and aerosol inhalation).
4. Regulatory and ethical issues also need to be addressed. CMV-derived products are between biological source materials and engineered medical devices, and the existing regulatory system is not perfect. Regulators can learn from the grading of cell therapy and the indication-specific approval path, and at the same time use the real-time review mechanism of mRNA vaccines to accelerate product deployment in the event of a sudden epidemic or an outbreak of drug-resistant bacteria. At the same time, vesicles from human or animal sources also need to establish traceable source and ethical norms.

Last but not least, cross-disciplinary integration will accelerate maturation. AlphaFold-assisted modelling can map toxin-receptor interfaces, guiding vesicle design; deep-learning interrogation of clinical multi-omics data will permit patient-specific formulation. Synthetic biology can programme producer cells to secrete vesicles with predefined functions, and self-assembling nano-clusters could create three-dimensional “immune-activating micro-domains” for sustained drug release. Ultimately, CMVs-derived formulations are poised to transcend single-disease tools, becoming a nexus between infectious disease science, immunology, materials science and clinical medicine. Continued innovation will unlock their full potential for precision therapy, outbreak control and personalized medicine, offering decisive solutions to antimicrobial resistance and immune imbalance.

## 5 Conclusion

This review systematically summarizes the bidirectional mechanisms of CMVs in the interaction between pathogens and hosts from the core perspective of “offense-defense game”, and focuses on the application potential of CMVs-derived formulations in biomimetic nanostrategies. This review, which differs from previous reviews that only focused on a single vesicle type or were limited to pathogenic mechanisms, provides a new framework for understanding the integrated role of CMVs-derived formulations in infection and immune regulation.

Future research needs to focus on three aspects: first, in-depth revelation of the dynamic role of CMVs in immune balance to clarify when to promote defense and when to lead to immune escape; second, optimization of vesicle engineering and production processes to solve the transformation bottlenecks such as batch differences, toxic residues and quality control; third, exploration of a wider range of clinical application scenarios, including the prevention and control of drug-resistant bacterial infections, immunotherapy and tumor-related applications. We believe that with the dual advancement of mechanism research and engineering technology, CMVs-derived formulations will provide a new breakthrough for the next generation of precision antibacterial and immunomodulatory strategies.
